# Supplementary material for: Differential behaviour of a risk score for emergency hospital admission by demographics in Scotland—A retrospective study
Source: PLOS Digit Health. 2024 Dec 17;3(12):e0000675. doi: 10.1371/journal.pdig.0000675 (PMC11651550; doi:10.1371/journal.pdig.0000675)
Supplement: S1 Appendix — This appendix contains details on the SPARRA score, on metrics used in this paper, and on analytical decisions. (PDF) [file pdig.0000675.s001.pdf]

# Differential behaviour of a risk score for emergency hospital admission by demographics in Scotland — a retrospective study

## Supplementary Appendix

Ioanna Thoma[1,2], Simon Rogers[4,5], Jillian Ireland[4], Rachel Porteous[4], Katie Borland[4], Catalina A. Vallejos[1,2*], Louis J. M. Aslett[1,3*], James Liley[1,3*]

**1** Alan Turing Institute, London, UK
**2** MRC Human Genetics Unit, Institute of Genetics and Cancer, University of Edinburgh, UK
**3** Department of Mathematical Sciences, Durham University, UK
**4** Public Health Scotland (PHS)
**5** School of Computing Science, University of Glasgow, UK

* Corresponding authors: james.liley@durham.ac.uk; catalina.vallejos@ed.ac.uk; louis.aslett@durham.ac.uk

# Contents


**1 Notation** **3**

**2 The SPARRA score** **3**

**3 Choice of patients on whom to intervene** **4**

**4 Score distribution** **6**
- 4.1 Demographic parity . . . . . . . . . . . . . . . . . . . . . . . 6
- 4.2 Counterfactual fairness . . . . . . . . . . . . . . . . . . . . . 6

**5 False negatives** **9**
- 5.1 Raw False Omission Rate . . . . . . . . . . . . . . . . . . . . 9
- 5.2 Adjusted FOR . . . . . . . . . . . . . . . . . . . . . . . . . . 9
- 5.3 Adjusted FOR as counterfactual . . . . . . . . . . . . . . . . 10
- 5.4 Outcome disparity . . . . . . . . . . . . . . . . . . . . . . . 12

**6 False positives** **12**

**7 Overall accuracy** **13**
- 7.1 ROC curves . . . . . . . . . . . . . . . . . . . . . . . . . . . 13
- 7.2 Calibration curves . . . . . . . . . . . . . . . . . . . . . . . 13

**8 Other metrics of fairness** **13**

**9 Discrimination and calibration** **14**

**10 Determination of ethnicity** **14**

# 1 Notation

We firstly consider 'covariates' which are attributes of individuals. We differentiate covariates as follows:

$U$ : all covariates; everything we know about an individual. Age, sex, SIMD, previous hospital activity data, prescriptions, ethnicity, urban-rural status. Does not include: specific postcode, marital status, smoking status, LGBT status, self-ID gender;

$X \subset U$ : covariates used in SPARRA; age, sex, SIMD, previous hospital activity data, prescriptions. Does not include ethnicity, mainland-island status or urban-rural status;

$Z = U \setminus X$ : all covariates *not* in $X$;

$A \subset X$ : age, sex, SIMD; covariates whose effect we will attempt to either isolate 'adjust away'.

We define the outcome value $Y$ for each individual as a Bernoulli random variable indicating whether that individual was admitted to hospital in the following year (1 if admitted, 0 if not). We model covariate and outcome values $(U, Y)$ for each individual as independent and identically distributed random variables with distribution $(U, Y) \sim \mathscr{D}$. All probabilities and expectations are over $\mathscr{D}$ unless otherwise specified. We view the SPARRA score as a fixed deterministic function $\hat{Y} = \hat{Y}(X) \in (0, 100)$.

In general, fairness metrics concern *a decision rule made on the basis of a risk score* rather than *the risk score itself* (e.g. [1, 2, 3]). Our effective decision rule is to take some action if the event $\{\hat{Y} \geq c\}$ occurs, and not take that action otherwise. We will be concerned with individuals in various groups defined by values of $U$. We will generally use the notation $G$ to indicate group membership (e.g., male sex, non-white ethnicity, island postcode). We presume $G$ can be derived from $U$, so we will use $G(U)$ where appropriate.

We will denote observations in our true data by $\{(u_i, \hat{y}_i)\}$, $i \in 1 \dots n$, with $u_i$ associated with $x_i, z_i, g_i$.

We will denote by $f_X(x)$ the PDF of a continuous random variable $X$ evaluated at $x$. We will generally treat random variables as continuous by default, though discrete analogs of statements are straightforward.

# 2 The SPARRA score

Our study uses the third version of the SPARRA score. The score comprises four logistic regression models, each used on a subgroup of the Scottish population: FE ('Frail elderly' cohort; individuals aged $\geq 75$), LTC ('Long-term conditions'; individuals aged 16-75 with prior healthcare system contact), YED ('Young emergency department'; individuals aged 16-55 who have had at least one A&E attendance in the previous year) and U16 ('under-16'; individuals

aged $< 16$). These groups cover approximately 80% of the Scottish population. For individuals present in both the LTC and YED subgroups, the maximum of the two risk prediction scores is reported. Input features include age, sex, an index of socioeconomic deprivation (using the deciles of the 2016 Scottish Index of Multiple Deprivation (SIMD) [4] as a geographic-based proxy), as well as information about long-term conditions, past hospital activity data and prescriptions. All features were derived from national electronic healthcare records (EHR) databases held by PHS, considering information up to three years prior to the prediction time cutoff (except for long-term conditions, which are extracted from historic records since 1981). SPARRAv3 scores were calculated by PHS and provided as input for our analysis. SPARRAv3 predicts emergency in-patient admission within 12 months from a given time cutoff. When training the model, individuals who died before the time cutoff or within 12 months after were excluded. In this analysis, rather than excluding individuals who died after the time cutoff, we considered a composite outcome of death or EA in the 12 months following the time cutoff. Since death indicates a poor health outcome that would otherwise be excluded from analysis, we can broadly consider SPARRAv3 as a tool for predicting abrupt breakdowns in health more generally [5].

We also repeated the analysis using SPARRAv4 [5], which is expected to be deployed in Scotland in 2024. SPARRAv4 was trained using more recent versions (2013-2018) of the same input data sources as SPARRAv3 and using more complex machine learning methods (e.g. random forests, gradient-boosted trees). This improved accuracy over SPARRAv3, though scores remained broadly similar [5]. To avoid duplication, this manuscript only presents results based on SPARRAv3. Other results can be explored on our interactive web application.

## 3 Choice of patients on whom to intervene

Suppose we are a practitioner with a set of patients for whom we have a well-calibrated risk score giving their probability of emergency hospital admission in the coming year. We have the resources to intervene on only a fixed number of patients, and we wish to choose these patients so as to find as many as possible who would go on to have an admission.

Intuitively, we would expect that the best way to choose this subset of patients would be to target those patients with risk scores exceeding some threshold. We will show that this intuition is correct, and that, although the risk score may exhibit different behaviour across subgroups of patients, we are best not to change this action threshold across groups.

When we say our risk score $\hat{Y} = \hat{Y}(X)$ is well-calibrated, we mean that it satisfies:

$$\hat{Y}(x) = P_{\mathscr{D}}(Y|X = x)$$

that is, it accurately estimates the risk of admission for an individual with

covariates $X = x$. If we say it is well-calibrated in a group $g$, we mean that:

$$\hat{Y}(x) = P_{\mathscr{D}}(Y|X = x, G = g)$$

Denote by $\mathscr{X}$ the domain of $X$, corresponding to the range of values covariates can take, where $X$ is a random variable with measure $\mu$ (so for a set of potential covariate values $X'$, $\mu(X')$ is the frequency at which patients have covariates in $X'$). We wish to choose a range of possible covariate values on which we will intervene. Due to cost constraints, we can only intervene on a fixed proportion of individuals.

Thus we wish to choose a region $\Gamma \subset \mathscr{X}$ with fixed measure $\gamma = \mu(\Gamma)$, optimising some objective. If we simply wish to choose $\Gamma$ so as to maximise the objective $P(Y|X \in \Gamma)$, then we should choose $\Gamma = \{x : \hat{Y}(x) > c\}$ for some $c$, since if $\Gamma' \neq \Gamma$ but $\mu(\Gamma) = \mu(\Gamma')$ and $\mu(\Gamma' \setminus \Gamma) > 0$ we have

$$\begin{aligned}
P(Y|X \in \Gamma) - P(Y|X \in \Gamma') &= \int_{\Gamma} \hat{Y} d\mu - \int_{\Gamma'} \hat{Y} d\mu \\
&= \int_{\Gamma \setminus \Gamma'} \hat{Y} d\mu - \int_{\Gamma' \setminus \Gamma} \hat{Y} d\mu \\
&> \int_{\Gamma \setminus \Gamma'} c d\mu - \int_{\Gamma' \setminus \Gamma} c d\mu \\
&= c \left( \int_{\Gamma \setminus \Gamma'} d\mu - \int_{\Gamma' \setminus \Gamma} d\mu \right) \\
&= c \left( \int_{\Gamma} d\mu - \int_{\Gamma'} d\mu \right) \\
&= 0
\end{aligned}$$

that is, if a practitioner wishes only to choose a subset of patients on whom to intervene such that they have the highest expected frequency of admissions, they should simply choose those patients for whom the SPARRA score exceeds some threshold. Intuitively, if we start with a group of individuals for which the SPARRA score exceeds a threshold $c$, and we change the group by removing someone and replacing them with someone else, then since the person we removed has a SPARRA score greater than $c$ and the person who replaced them has a score less than $c$, we are necessarily reducing the expected number of people we find who will eventually be admitted.

Importantly, this is independent of any group structure: if we have the option of varying the threshold between groups, if we simply aim to 'catch' as many individuals as possible with $Y = 1$, we should use an identical threshold on $\hat{Y}$ in all groups. We consider groupings defined by values of a random variable $G$. Because the score is well-calibrated for all groups, we have:

$$P(Y|G = g, \hat{Y}(X) = y) = y = P(Y|\hat{Y}(X) = y)$$

so $Y \perp\!\!\!\perp G|\hat{Y}$; or equivalently, $G$ does not contribute further information to knowledge of the probability of $Y = 1$ once we know $\hat{Y}(X)$. Hence the optimality

of $\Gamma$ does not depend on our groupings, and a practitioner aiming to target patients so as to target as many admissions as possible in total should target individuals with score in excess of a given threshold, where the threshold is identical over groups, regardless of the distribution of scores in those groups.

In particular, even if a score is well-calibrated in two groups $G = g$ and $G = g'$, use of a single threshold $c$ in a decision rule 'act if $\hat{Y} > c$' may lead to differential frequencies of the event $Y = 1$ in both groups; that is:

$$P(Y = 1|G = g, \hat{Y} < c) \neq P(Y = 1|G = g', \hat{Y} < c) \,.$$

# 4 Score distribution

## 4.1 Demographic parity

We firstly assess demographic parity [6, 7]: the distribution of scores across groups, termed $DP$, defined as

$$DP(c, g) = P(\hat{Y} \geq c|G = g)$$

across cutoffs $c$ and groups $g$. We use the standard (uniformly consistent) CDF estimator:

$$DP(c, g) \approx \frac{|\{i : \hat{y}_i \geq c, g_i = g\}|}{|\{i : g_i = g\}|} \tag{1}$$

## 4.2 Counterfactual fairness

We then compute counterfactual fairness between groups, which can be thought of as describing differences in distribution of score due only to the effect of group on (some of) age, sex, and deprivation and directly on the score. We do this by considering a hypothetical individual who resembles a typical member of a given group, except that they are a member of another different group. This requires the specification of a system of causality relating covariates, group, and predicted score. We use the following causal model:

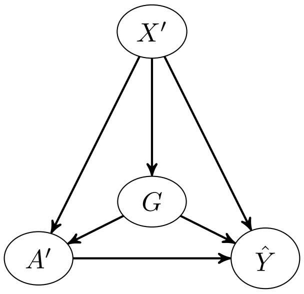


In this graph, vertex $G$ denotes group (e.g. urban/rural, older/younger). Vertex $A'$ denotes $(A \setminus G)$; that is, age, sex and SIMD, except any of these

which determine $G$. Vertex $X'$ denotes $(X \setminus (A \cup G))$; that is, covariates in SPARRA score excepting age, sex, SIMD and variable determining $G$. Vertex $\hat{Y}$ denotes the prediction from SPARRA score. The distribution of *background variable* $X'$ is its marginal distribution in $\mathscr{D}$.

The *structural equation* for vertex $V$ with parents $P$ is denoted $s^1_P(v;p)$ and is given by $s^1_P(v;p) = P_{\mathscr{D}}(V = v|P = p)$, substituting densities for probabilities where appropriate. For instance, the structural equation for $A$ is $s^1_{A'}(a';g,x') = P_{\mathscr{D}}(A' = a'|G = g, X = x')$. The superscript '1' identifies the structural equation with this causal graph as opposed to later causal graphs.

We compare the counterfactual values, which we term $DPC$:

$$DPC(c,g,g') = P(\hat{Y}_{G \leftarrow g'} \geq c|G = g) \tag{2}$$

between cutoffs $c$, and for either $g' = g$ or $g' \neq g$. We compute values (2) in three steps [8, 9]:

1. Compute the posterior distribution $\mathscr{X}' \sim (X'|G = g)$; that is, the distribution of everything other than age, sex, SIMD for members of $g$.
2. Delete the edge from $X'$ to $G$ and set $G = g'$; that is ignore the influence of non-age, sex, SIMD covariates on group status, and presume we are dealing with a member of $g'$,
3. Compute the joint distribution $(\mathscr{X}', \mathscr{A}')$ with density at $x', a'$ given by

$$f_{(\mathscr{X}', \mathscr{A}')}(x', a') = s^1_{A'}(a';g,x') f_{\mathscr{X}'}(x') \tag{3}$$

   that is, compute the distribution of non-group covariates for a person whose group is $g$, but for which age, sex and SIMD are distributed as though they were a random member of the population with $\hat{Y} < c$,

4. Set random variable $(\hat{Y}_{G \leftarrow g'}|G = g)$ with density at $y$ given by

$$f_{\hat{Y}_{G \leftarrow g'}|G=g}(y) = \mathbb{E}_{(X',A') \sim (\mathscr{X}', \mathscr{A}')} \left\{ s^1_{\hat{Y}}(y; X', A', g') \right\} \tag{4}$$

   that is, compute the distribution of predicted score of a person for which all non-age, sex, SIMD covariates are distributed as though they were in group $g$, but they are in group $g'$ and have corresponding values of age, sex, and SIMD.

We sample from the distribution of $(\hat{Y}_{G \leftarrow g'}|G = g)$ as follows:

1. Choose a random individual with $G = g$, and note their values $x'$ of $X'$
2. Choose a random individual with $G = g'$ for whom $X = x'$
3. Record the value of $\hat{Y}$ for this individual as a sample from $(\hat{Y}_{G \leftarrow g'}|G = g)$

To see why this works, we note that in step 2 we are sampling a random individual amongst the population with $X = x'$ and $G = g'$; hence the distribution $\hat{Y}$ we obtain in step 3 conditional on our choice of $x'$ in step 2 is $(\hat{Y}|G = g', X = x')$. In step 1, we are choosing a value of $X'$ from the distribution $(X'|G = g)$. The distribution of $\hat{Y}$ values arising from the procedure is hence given by $f_{samp}(y)$ where:

$$\begin{aligned} f_{samp}(y) &= \mathbb{E}_{x' \sim (X'|G=g)} \left\{ f_{\hat{Y}|G=g',X=x'}(y) \right\} \\ &= \int f_{\hat{Y}|G=g',X=x'}(y) f_{X'|G=g}(x') dx' \end{aligned}$$

Working from the definition of $(\hat{Y}_{G \leftarrow g'}|G = g)$ we have

$$\begin{aligned} f_{\hat{Y}_{G \leftarrow g'}}(y) &= \mathbb{E}_{(X',A') \sim (\mathscr{X}',\mathscr{A}')} \left\{ s^1_{\hat{Y}}(y; X', A') \right\} \\ &= \iint s^1_{\hat{Y}}(y; x', a', g') f_{(\mathscr{X}',\mathscr{A}')}(x', a') dx' da' \\ &= \iint s^1_{\hat{Y}}(y; x', a') s^1_{A'}(a'; g', x') f_{\mathscr{X}'}(x') dx' da' \\ &= \iint f_{\hat{Y}|G=g',X=x',A=a'}(y) f_{A'|G=g',X=x'}(a') da' f_{X'|G=g}(x') dx' \\ &= \int f_{\hat{Y}|G=g',X=x'}(y) f_{X'|G=g}(x') dx' \\ &= f_{samp}(y) \end{aligned}$$

as required. For group $g$, rather than choosing a random individual in step 1, we run through all samples $i$ with $g_i = g$, producing $|\{i : g_i = g\}|$ corresponding samples of $(\hat{Y}_{G \to g'}|G = g)$. We estimate quantity 2 analogously to the estimate (1) with the estimates of $(\hat{Y}_{G \to g'}|G = g)$ in place of the $y_i$. We provide a general implementation of this procedure in our R package.

Formal counterfactual fairness [8] requires the identity:

$$P(\hat{Y}_{G \leftarrow g'} \geq c | G = g, X' = x') = P(\hat{Y}_{G \leftarrow g} \geq c | G = g, X' = x') \tag{5}$$

to hold for all values $x'$, $g$ and $g'$; we only compare the mean quantities:

$$\begin{aligned} DPC(c, g, g') &= P(\hat{Y}_{G \leftarrow g'} \geq c | G = g) \\ &= \int P(\hat{Y}_{G \leftarrow g'} \geq c | G = g, X' = x') f_{X'}(x') dx \end{aligned}$$

where $f_{X'}(x')$ is the marginal density of $X'$ in the common distribution of $(U, Y)$.

The comparison of counterfactuals between groups amounts to assessment of the difference in distribution of $\hat{Y}$ attributable only to effects of group on $A'$. Broadly, we are interested in the two classes of causes of variation in $\hat{Y}$: $A'$ and $X'$. We view $X'$ (hospital activity data, prescriptions, long term conditions) as 'underlying' causes of variation in $\hat{Y}$, where variables in $A'$ act as 'modulators' to the score. We model this with an edge $X'$ to $A'$.

We note that groups $G$ are associated with different posteriors over $X$, so we model an edge $X'$ to $G$. We wish to include the effects of group $G$ on the 'modulation' of the effect of $X'$ on $Y$ through $A'$ so include an edge from $G$ to $A'$, but discount the variation in posteriors in 'legitimate' covariates $X'$. The counterfactual $(\hat{Y}_{G \leftarrow g'}|G = g)$ achieves this by assessing the difference in $\hat{Y}$ between that arising from the full causal graph and the causal graph with the $X' \to G$ edge removed, while choosing the background variable $X'$ to be distributed as though $G$ were $g$ in both cases.

# 5 False negatives

## 5.1 Raw False Omission Rate

We characterise 'false negative' errors using false omission rate (FOR), termed $FOR$ [3, 10]. We compare the values of

$$FOR(c, g) = P(Y = 1|G = g, \hat{Y} < c) \tag{6}$$

across cutoffs $c$ and groups $g$. The events $Y = 1$, $\hat{Y} < c$ indicate in a sense that the prediction $\hat{Y} < c$ was incorrect. The FOR indicates a measure of how often individuals in a group predicted *not* to be admitted were in fact admitted. We estimate FOR consistently as

$$FOR(c, g) \approx \frac{|\{i : y_i = 1, \hat{y}_i < c, g_i = g\}|}{|\{i : \hat{y}_i < c, g_i = g\}|} \tag{7}$$

## 5.2 Adjusted FOR

We also consider FOR 'adjusted' for variables $A' = \{A \setminus G\}$ (age, sex, and deprivation, excluding any variables that determine $G$). We denote

$$FOR_{a'}(c, g) = P(Y = 1|G = g, \hat{Y} < c, A' = a')$$

from which we may write quantity (6) as:

$$\begin{aligned} FOR(c, g) &= P(Y = 1|G = g, \hat{Y} < c) \\ &= \int P(Y = 1|G = g, A = a', \hat{Y} < c) f_{(A'|G=g,\hat{Y}<c)}(a')da' \\ &= \int FOR_{a'}(c, g) f_{(A|G=g,\hat{Y}<c)}(a')da' \end{aligned}$$

To adjust for $A'$, we replace the density $f_{(X|G=g,\hat{Y}<c)}(x)$ with $f_{A'|\hat{Y}<c}(a')$, which does not depend on $g$. This generates adjusted FOR, which we term $FOR_A$:

$$FOR_A(c, g) = \int FOR_{a'}(c, g) f_{A'|\hat{Y}<c}(a')da' \tag{8}$$

Our data discretises $A'$ (rounding age to the nearest year). Writing $\mathbb{A}$ as the set of possible discrete $A'$ values, we estimate $FOR_A(c, g)$ as:

$$\begin{aligned} FOR_A(c,g) &\approx \sum_{a' \in \mathbb{A}} FOR_{a'}(c,g) P(A' = a' | \hat{Y} < c) \\ &\approx \sum_{a' \in \mathbb{A}} \frac{|\{i : y_i = 1, a_i = a', \hat{y}_i < c, g_i = g\}|}{|\{i : a_i = a', \hat{y}_i < c, g_i = g\}|} \frac{|\{i : a_i = a', \hat{y}_i < c\}|}{|\{i : \hat{y}_i < c\}|} \end{aligned} \tag{9}$$

where the first term in the sum is an estimate of $FOR_{a'}(c, g)$ and the second is an estimate of $P(A' = a'|\hat{Y} < c)$. The second approximation is consistent.

## 5.3 Adjusted FOR as counterfactual

The quantity $FOR_A(c, g)$ can be viewed as a comparison of counterfactual quantities

$$P_{\mathscr{D}|\hat{Y}<c}(Y_{G \leftarrow g'} = 1) \tag{10}$$

across groups $g$, under the causal specification as follows:

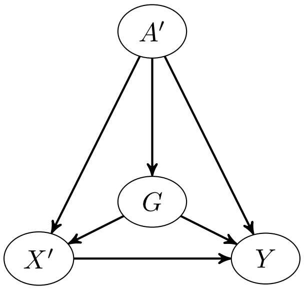


Note $X'$ and $A'$ are swapped from their positions in the previous causal graph. Since we are restricting entirely to individuals for which $\hat{Y} < c$, we emphasise that all probabilities in the background variables and structural equations are with respect to the distribution $\mathscr{D}|\hat{Y} < c$ rather than $\mathscr{D}$. Vertices $G$, $X'$ and $A'$ have the same meanings as in Section 4.2; vertex $Y$ indicates outcome; that is, admission or non-admission in coming year. The *background variable* $A'$ has distribution given by the marginal of $A'$ in $\mathscr{D}|\hat{Y} < c$. Similarly to the previous causal graph, we denote the *structural equation* of vertex $V$ with parents $P$ as $s^2_V(v; p)$ given by $s^2_V(v, p) = P_{\mathscr{D}|\hat{Y}<c}(V = v|P = p)$.

To see the equivalence of quantities (8) and (10), note that the analogous procedure to counterfactual fairness above computes $Y_{G \leftarrow g'}$ as:

1. Denote by $\mathscr{A}'$ the distribution of $A'|\hat{Y} < c$, or equivalently the marginal of $A'$ in $\mathscr{D}|\hat{Y} < c$; that is, the distribution of age, sex and SIMD over the whole population.

2. Delete the edge from $A'$ to $G$ in the graph and set $G = g$,; that is ignore the influence of non-age, sex, SIMD covariates on group status, and presume we are dealing with a member of $g$ whose values of $A'$ are sampled as though they were a random member of the population with $\hat{Y} < c$.
3. Compute the joint distribution $(\mathscr{X}', \mathscr{A}')$ with density at $x', a'$ given by $f_{(\mathscr{X},\mathscr{A})}(x', a') = s^2_{X'}(x; g, a') f_{\mathscr{A}}(a')$; that is, compute the distribution of non-group covariates for a person whose group is $g$, but for which age, sex and SIMD are distributed as though they were a random member of the population with $\hat{Y} < c$,
4. Now $P_{\mathscr{D}|\hat{Y}<c}(Y_{G\leftarrow g} = y) = \mathbb{E}_{(X',A')\sim(\mathscr{X}',\mathscr{A}')} \left\{ s^2_Y(y; X', A', g) \right\}$; that is, we compute the distribution of predicted score of a person for which age, sex, and SIMD are distributed as though they were a random member of the population, but for which their group is $g$.

Now

$$\begin{aligned}
P_{\mathscr{D}|\hat{Y}<c}(Y_{G\leftarrow g} = 1) &= \mathbb{E}_{(X',A')\sim(\mathscr{X},\mathscr{A})} \left\{ s^2_Y(1; X', A', g) \right\} \\
&= \iint s^2_Y(1; x', a', g) f_{(\mathscr{X},\mathscr{A})}(x', a') dx' da' \\
&= \iint s^2_Y(1; x', a', g) s^2_{X'}(x'; g, a') f_{\mathscr{A}}(a') dx' da' \\
&= \iint P(Y = 1|G = g, X' = x', \hat{Y} < c, A = a') \cdot \\
&\qquad P(X' = x'|G = g, \hat{Y} < c, A = a') dx' f_{A'|\hat{Y}<c}(a') da' \\
&= \iint P(Y = 1, X' = x'|G = g, \hat{Y} < c, A = a') dx' f_{A'|\hat{Y}<c}(a') da' \\
&= \int P(Y = 1|G = g, \hat{Y} < c, A = a') f_{A'|\hat{Y}<c}(a') da' \\
&= \int FOR_{a'}(c, g) f_{A'}(a') da' \\
&= FOR_A(c, g)
\end{aligned}$$

In contrast to $DPC$, the counterfactual in this case adjusts away for the effect of group $G$ on demographics $A'$ while including the effects of $G$ on $Y$. Correspondingly, the causal graph in this case has reversed positions of $X'$, $A'$ compared with the causal graph in Section 4.2

An alternative measure of false negatives would be to measure the false negative rates directly; for instance, using $FNP$ which compares

$$FNP(c, g) = P(\hat{Y}^c = 0, Y = 1|G = g) \tag{11}$$

between groups $g$ and across cutoffs $c$. We chose $FOR$ ahead of $FNP$ because the population of individuals of interest to GPs is better represented by the

individuals for which $G = g, \hat{Y}^c = 0$ (the conditional population in $FOR$) than the population with just $G = g$ (the conditional population in $FNP$), in that the former represents individuals with $G = g$ judged as low-risk by SPARRA. We compare $FNP(c, g)$ between groups $g$ in the Supplementary Figures and in the Shiny app associated with this manuscript.

## 5.4 Outcome disparity

Individuals in the set of the numerator of the $FOR(c, g)$ estimate (equation 7) were all admitted to hospital ($y_i = 1$) despite being 'predicted not to' ( $\hat{y}_i < c$). Most individuals admitted to hospital have a recorded primary diagnosis, coded according to the ICD10 criteria [11]. We considered the distribution of the first letter of these ICD10 codes across individuals, giving a breakdown of admission causes amongst admitted individuals with $\hat{y}_i < c$ (S1 Table).

Denote admission causes by a random variable $W$ (with observations $w_i$). For an admission cause $w$, group $g$, and fixed score cutoff $c = 0.1$, we make the estimates

$$A = \frac{n_A}{d_A} = \frac{|\{i : w_i = 1, y_i = 1, g_i = g\}|}{|\{i : y_i = 1, g_i = g\}|} \approx P(W = w|G = g, Y = 1)$$
$$B = \frac{n_B}{d_B} = \frac{|\{i : g_i = g, w_i = 1, y_i = 1, \hat{y}_i < c\}|}{|\{i : y_i = 1, g_i = g, \hat{y}_i < c\}|} \approx P(W = w|G = g, Y = 1, \hat{Y} < c)$$

and plot $A$ against $B - A$ (see Fig 3 in main paper). We estimate standard errors of $B - A$ as:

$$SE(B - A) \approx \sqrt{\frac{A(1 - A)}{n_A} + \frac{B(1 - B)}{n_B}}$$

and confidence intervals through these standard errors.

# 6 False positives

We characterise 'false positive' errors analogously to false-negative errors using false-discovery rate parity, termed FDRP [10], defined as

$$FDRP(c, g) = P(Y = 0|G = g, \hat{Y} \geq c) \quad (12)$$

and again compute the 'adjusted' version of this quantity, defined as:

$$FDRP_{a'}(c, g) = P(Y = 0|G = g, \hat{Y} \geq c, A = a')$$
$$FDRP_A(c, g) = \int FDRP_{a'}(c, g) f_{A'|\hat{Y} \geq c}(a') da'$$

which can be viewed as a comparison of counterfactual quantities

$$P_{\mathscr{D}|\hat{Y} \geq c}(Y_{G \leftarrow g'} = 0)$$

across groups $g$, under the causal specification in Section 5, noting that probabilities are with respect to the distribution $(\mathscr{D}|\hat{Y} \geq c)$ rather than $(\mathscr{D}|\hat{Y} < c)$. We estimated these in an analogous way to $FOR(c,g)$ and $FOR_A(c,g)$ (equations (7) and (9)

# 7 Overall accuracy

## 7.1 ROC curves

We firstly analyse group-level accuracy in each group using discrimination (area under receiver-operator characteristic, AUROC) and calibration. The AUROC (also called the C-statistic) estimates

$$AUROC(g) = P\left(\hat{Y}(X_1) > \hat{Y}(X_2)|Y_1 > Y_2, G(U_1) = G(U_2) = g\right)$$

over groups $g$, where $(U_1, Y_1) = (X_1, Z_1, Y_1)$ and $(U_2, Y_2) = (X_2, Z_2, Y_2)$ have the common distribution of $(U, Y)$. That is, the AUROC estimates the probability that the score for a randomly chosen admitted individual exceeds the score for a randomly chosen non-admitted individual. We use the (consistent) estimator:

$$AUROC(g) \approx \frac{|\{(i,j) : \hat{y}_i > \hat{y}_j, y_i > y_j, g_i = g_j = g\}|}{|\{(i,j) : y_i > y_j, g_i = g_j = g\}|} \tag{13}$$

## 7.2 Calibration curves

We secondly analyse group-level accuracy by means of calibration curves (also called reliability diagrams) [12] in which for a range of cutoffs $c$ we compare the difference of $P(Y|\hat{Y} = c)$ from $c$: that is, whether individuals with a score of $c$ are admitted in on average $c\%$ of cases.

We consider the intervals $I_h = [\frac{h-1}{10}, \frac{h}{10})$ for $h \in 1:10$. For each interval we make the (consistent) estimates:

$$\mathbb{E}\{Y|\hat{Y} \in I_h\} \approx \frac{\sum_{i:\hat{y}_i \in I_h} y_i}{|\{i : \hat{y}_i \in I_h\}|}$$

$$\mathbb{E}\{\hat{Y}|\hat{Y} \in I_h\} \approx \frac{\sum_{i:\hat{y}_i \in I_h} \hat{y}_i}{|\{i : \hat{y}_i \in I_h\}|}$$

with confidence intervals and standard errors estimated using the usual asymptotic confidence interval of a proportion. We plot these estimates directly for comparison.

# 8 Other metrics of fairness

We also compute the following metrics for group fairness, available on our Shiny app. These are all compared between groups $g$ at a range of cutoffs $c$.

$$FPP(c,g) : \text{False positive parity: } P(\hat{Y}=1, Y=0|G=g)$$

$$FPRP(c,g) : \text{False positive rate parity: } P(\hat{Y}=1, Y=0|G=g)$$

$$RP(c,g) : \text{Recall parity: } P(\hat{Y}=1|G=g, Y=1)$$

$$IRP(c,g) : \text{Inverse recall parity: } P(\hat{Y}=0|G=g, Y=0)$$

$$FNP(c,g) : \text{False negative parity: } P(\hat{Y}=0, Y=1|G=g)$$

$$FNRP(c,g) : \text{False negative rate parity: } P(\hat{Y}=0|G=g, Y=1)$$

All are estimated using consistent estimators analogous way to that used for $FOR(c,g)$ (equation (7)). We provide general code to estimate all metrics in our R package.

# 9 Discrimination and calibration

In comparison with the overall cohort, the largest differences in discrimination (as measured by AUROC) were observed for the subgroups defined by age and ethnicity. Discrimination was stronger in individuals over 65 than in those younger than 25 (S3 Fig, Panel A), indicating that EAs are more readily predictable within this group. Although both nonwhite and white subgroups had poorer discrimination than the overall cohort (Panel D), we did not observe large differences in AUROC between these subgroups. Less prominent differences were observed in all other comparisons, where AUROC within each subgroup was similar to the overall AUROC: discrimination was slightly stronger in the rural than in urban residents (Panel E) and in females than in males (Panel B), but differences were almost negligible between mainland and island residents (Panel F).

Despite small deviations between the predicted and observed number of events, SPARRA is generally well-calibrated in the overall cohort, and within most of the subgroups considered in our analysis (S4 Fig). The largest departures from perfect calibration were observed for the subgroups defined by age and ethnicity. The estimated calibration curve suggests that SPARRA may overestimate risk for the younger group, although with a high degree of uncertainty attached (Panel A). Moreover, SPARRA also appears to underestimate EA risk for both white and nonwhite subgroups (Panel D).

# 10 Determination of ethnicity

Ethnicity data was determined for individuals in our dataset by Public Health Scotland by cross-reference of information across a range of sources. In particular, the following datasets were considered:

1. Records from the COVID-19 vaccination program from November 2021 onwards

2. Outpatient and inpatient or day case hospital records from March 2010 [13, 14]

3. Rapid Preliminary hospital Inpatient Data records from February 2020 [15]

4. Data from the COVID Case Management System from June 2020

5. Electronic Communication of Surveillance in Scotland from February 2020 [16]

6. Urgent Care Datamart from January 2011 Methodology [17, 18]

For each source of ethnicity, the CHI number (a national individual-level identifier), ethnicity identifier and date of capture were imported. The ethnicity identifier varies between data sources, so each lookup has its own mapping.

Ethnicity classifications are based on the Scottish 2011 Census categories [19] which are used as a standard across NHS Scotland.

Once ethnicity identifiers had been mapped records were removed where

1. CHI number is missing or appears malformed.

2. Ethnicity identifier could not be mapped.

All sources were joined and sorted by date of capture. Ethnicity records are considered valid for inclusion if any of the following were true:

1. The ethnicity did not come from the vaccination program

2. The ethnicity came from the vaccination program, but was added before 5th September 2022

When two or more ethnicity records were present for a given patient, the most recent ethnicity recorded for a patient was retained.

Our analysis revealed some differences between individuals with white and nonwhite recorded ethnicity. Among others, calibration was poorer for both groups than for the overall cohort, but better for white than nonwhite ethnic groups. However, the interpretation of these results is not straightforward due to high levels of non-random missingness for ethnicity.

## References


[1] Hardt M, Price E, Srebro N. Equality of opportunity in supervised learning. Advances in neural information processing systems. 2016;29.

[2] Corbett-Davies S, Pierson E, Feller A, Goel S, Huq A. Algorithmic decision making and the cost of fairness. In: Proceedings of the 23rd acm sigkdd international conference on knowledge discovery and data mining; 2017. p. 797–806.

[3] Baumann J, Hannák A, Heitz C. Enforcing Group Fairness in Algorithmic Decision Making: Utility Maximization Under Sufficiency. In: 2022 ACM Conference on Fairness, Accountability, and Transparency. FAccT '22. New York, NY, USA: Association for Computing Machinery; 2022. p. 2315–2326. Available from: https://doi.org/10.1145/3531146.3534645.

[4] Scottish Government. Scottish index of multiple deprivation; 2016.

[5] Liley J, Bohner G, Emerson SR, Mateen BA, Borland K, Carr D, et al. Development and assessment of a machine learning tool for predicting emergency admission in Scotland. medRxiv. 2021; p. 2021–08.

[6] Calders T, Kamiran F, Pechenizkiy M. Building classifiers with independency constraints. In: 2009 IEEE International Conference on Data Mining Workshops. IEEE; 2009. p. 13–18.

[7] Zliobaite I. On the relation between accuracy and fairness in binary classification. arXiv preprint arXiv:150505723. 2015;.

[8] Kusner MJ, Loftus J, Russell C, Silva R. Counterfactual fairness. Advances in neural information processing systems. 2017;30.

[9] Pearl J. Causal diagrams for empirical research. Biometrika. 1995;82(4):669–688.

[10] Saleiro P, Kuester B, Hinkson L, London J, Stevens A, Anisfeld A, et al. Aequitas: A bias and fairness audit toolkit. arXiv preprint arXiv:181105577. 2018;.

[11] World Health Organization. International statistical classification of diseases and related health problems. vol. 1. World Health Organization; 2004.

[12] Bröcker J, Smith LA. Increasing the reliability of reliability diagrams. Weather and forecasting. 2007;22(3):651–661.

[13] "Public health information Scotland". SMR00 - Outpatient attendance; 2020.

[14] "Public health information Scotland". SMR01 - Acute Hospital Admissions; 2020.

[15] "Public health information Scotland". System Watch: urgent care usage; 2020.

[16] "Public health information Scotland". Infection Intelligence Platform (IIP): High Level Guide to IIP Component Datasets held by NHS National Services Scotland; 2014.

[17] "Public health information Scotland". AE2 - accident and emergency records; 2020.

[18] "Public health information Scotland". SMR01 - Acute Hospital Admissions; SMR04 - Psychiatric Hospital Admissions; 2020.

[19] Office for National Statistics, National Records of Scotland, Northern Ireland Statistics and Research Agency. 2011 Census aggregate data. UK Data Service (Edition: June 2016); 2016.
