# Supplementary material for: Differential behaviour of a risk score for emergency hospital admission by demographics in Scotland—A retrospective study
Source: PLOS Digit Health. 2024 Dec 17;3(12):e0000675. doi: 10.1371/journal.pdig.0000675 (PMC11651550; doi:10.1371/journal.pdig.0000675)
Supplement: S1 Table — (PDF) [file pdig.0000675.s002.pdf]

| ICD10 code begins with: | Admission type |
|---|---|
| A;B | Infectious disease |
| C; D1;D2;D3;D4 | Neoplasm |
| D5;D6;D7;D8;D9 | Blood |
| E | Endocrine/metabolic |
| F | Mental/behavioural |
| G | Nervous system |
| H1;H2;H3;H4;H5 | Eye |
| H6;H7;H8;H9 | Ear |
| I | Circulatory |
| J | Respiratory |
| K | Digestive |
| L | Skin |
| M | Musculoskeletal |
| N | Genitourinary |
| O | Obstetric/puerperium |
| P | Perinatal |
| Q | Congenital |
| R | Abnormality NEC |
| S;T;V;X;Y | External |
| U;Z | Other |

**S1 Table. Definition of different admission types.**
