# Supplementary material for: Differential behaviour of a risk score for emergency hospital admission by demographics in Scotland—A retrospective study
Source: PLOS Digit Health. 2024 Dec 17;3(12):e0000675. doi: 10.1371/journal.pdig.0000675 (PMC11651550; doi:10.1371/journal.pdig.0000675)
Supplement: S1 Fig — Lower sub-panels on each panel show difference between curves. Coloured bands show pointwise 95% confidence intervals. Vertical red dashed lines identify a score of 10%. Figures for age and SIMD are replicated from the main text. (PDF) [file pdig.0000675.s003.pdf]

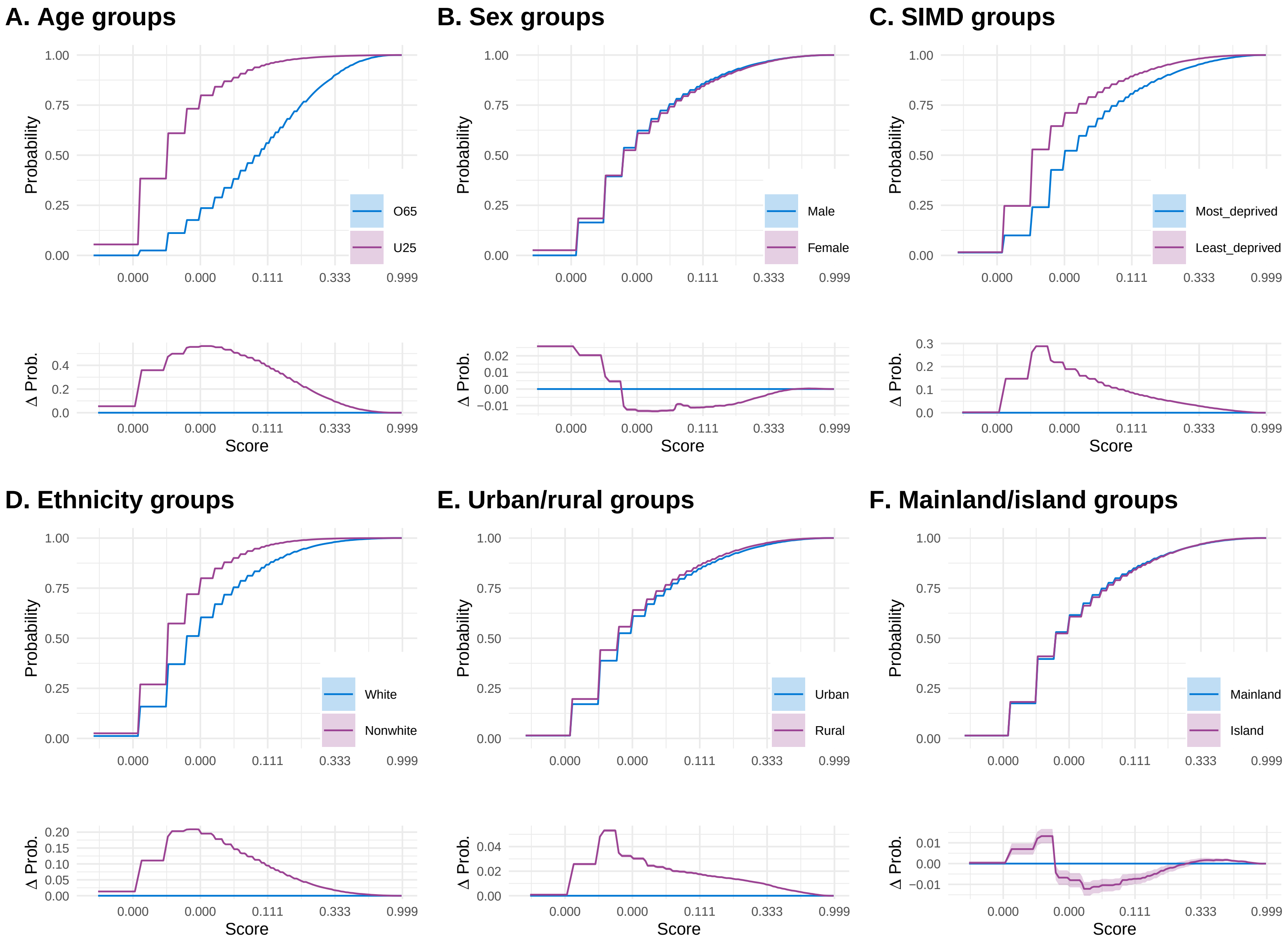

A. Age groups
Probability
1.00
0.75
0.50
0.25
0.00
O65
U25
0.000
0.000
0.111
0.333
0.999
Δ Prob.
0.4
0.2
0.0
Score
B. Sex groups
Male
Female
0.02
0.01
0.00
−0.01
C. SIMD groups
Most_deprived
Least_deprived
0.3
0.2
0.1
0.0
D. Ethnicity groups
White
Nonwhite
0.20
0.15
0.10
0.05
0.00
E. Urban/rural groups
Urban
Rural
0.04
0.02
0.00
F. Mainland/island groups
Mainland
Island
0.01
0.00
−0.01
