# Supplementary material for: Differential behaviour of a risk score for emergency hospital admission by demographics in Scotland—A retrospective study
Source: PLOS Digit Health. 2024 Dec 17;3(12):e0000675. doi: 10.1371/journal.pdig.0000675 (PMC11651550; doi:10.1371/journal.pdig.0000675)
Supplement: S3 Fig — Legends show AUROC, and associated standard errors. Lower sub-panels on each panel show difference between curves. Points on figures identify a score of 10%. (PDF) [file pdig.0000675.s005.pdf]

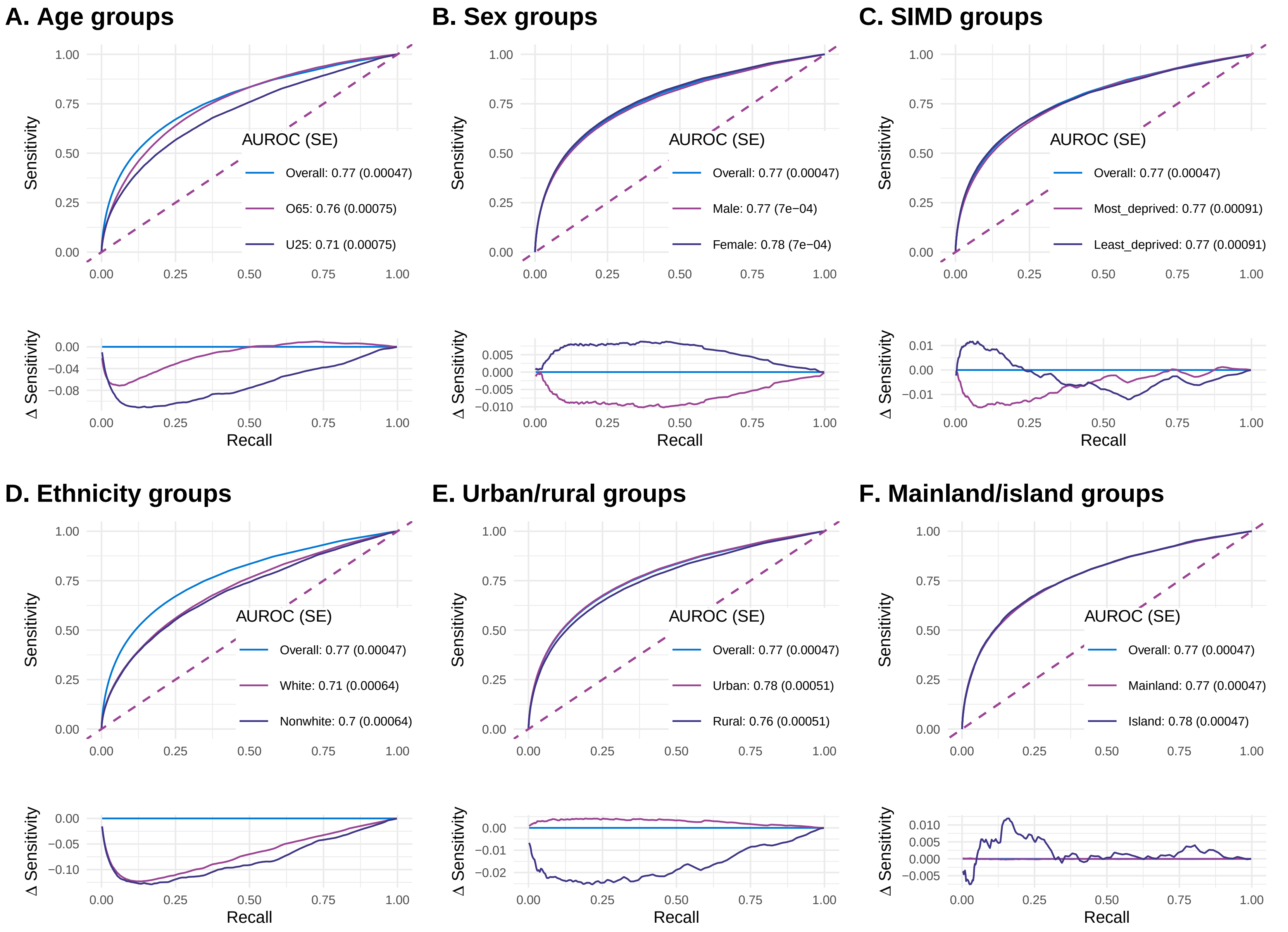

A. Age groups
B. Sex groups
C. SIMD groups
D. Ethnicity groups
E. Urban/rural groups
F. Mainland/island groups
Sensitivity
Δ Sensitivity
Recall
AUROC (SE)
Overall: 0.77 (0.00047)
O65: 0.76 (0.00075)
U25: 0.71 (0.00075)
Male: 0.77 (7e−04)
Female: 0.78 (7e−04)
Most_deprived: 0.77 (0.00091)
Least_deprived: 0.77 (0.00091)
White: 0.71 (0.00064)
Nonwhite: 0.7 (0.00064)
Urban: 0.78 (0.00051)
Rural: 0.76 (0.00051)
Mainland: 0.77 (0.00047)
Island: 0.78 (0.00047)
