# Supplementary material for: Differential behaviour of a risk score for emergency hospital admission by demographics in Scotland—A retrospective study
Source: PLOS Digit Health. 2024 Dec 17;3(12):e0000675. doi: 10.1371/journal.pdig.0000675 (PMC11651550; doi:10.1371/journal.pdig.0000675)
Supplement: S4 Fig — Lower sub-panels on each panel show difference between curves relative to whole group. Coloured bands indicate 95% pointwise confidence intervals. Points on figures identify a score of 10%. (PDF) [file pdig.0000675.s006.pdf]

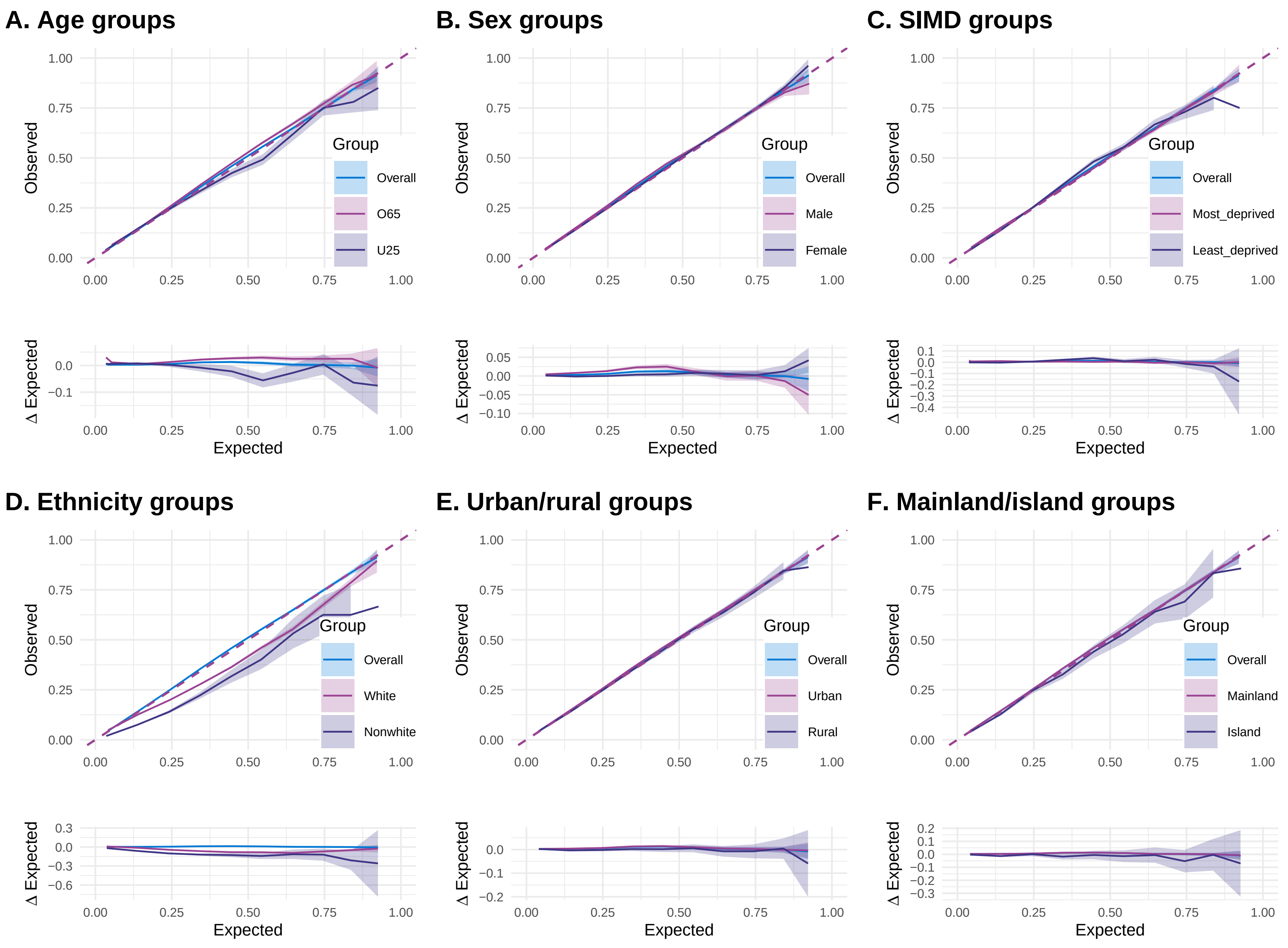
A. Age groups
B. Sex groups
C. SIMD groups
D. Ethnicity groups
E. Urban/rural groups
F. Mainland/island groups
Observed
Δ Expected
Expected
Group
Overall
O65
U25
Male
Female
Most_deprived
Least_deprived
White
Nonwhite
Urban
Rural
Mainland
Island
