# Supplementary material for: Differential behaviour of a risk score for emergency hospital admission by demographics in Scotland—A retrospective study
Source: PLOS Digit Health. 2024 Dec 17;3(12):e0000675. doi: 10.1371/journal.pdig.0000675 (PMC11651550; doi:10.1371/journal.pdig.0000675)
Supplement: S5 Fig — Lower sub-panels on each panel show difference between curves. Coloured bands show pointwise 95% confidence intervals. Vertical red dashed lines identify a score of 10%. (PDF) [file pdig.0000675.s007.pdf]

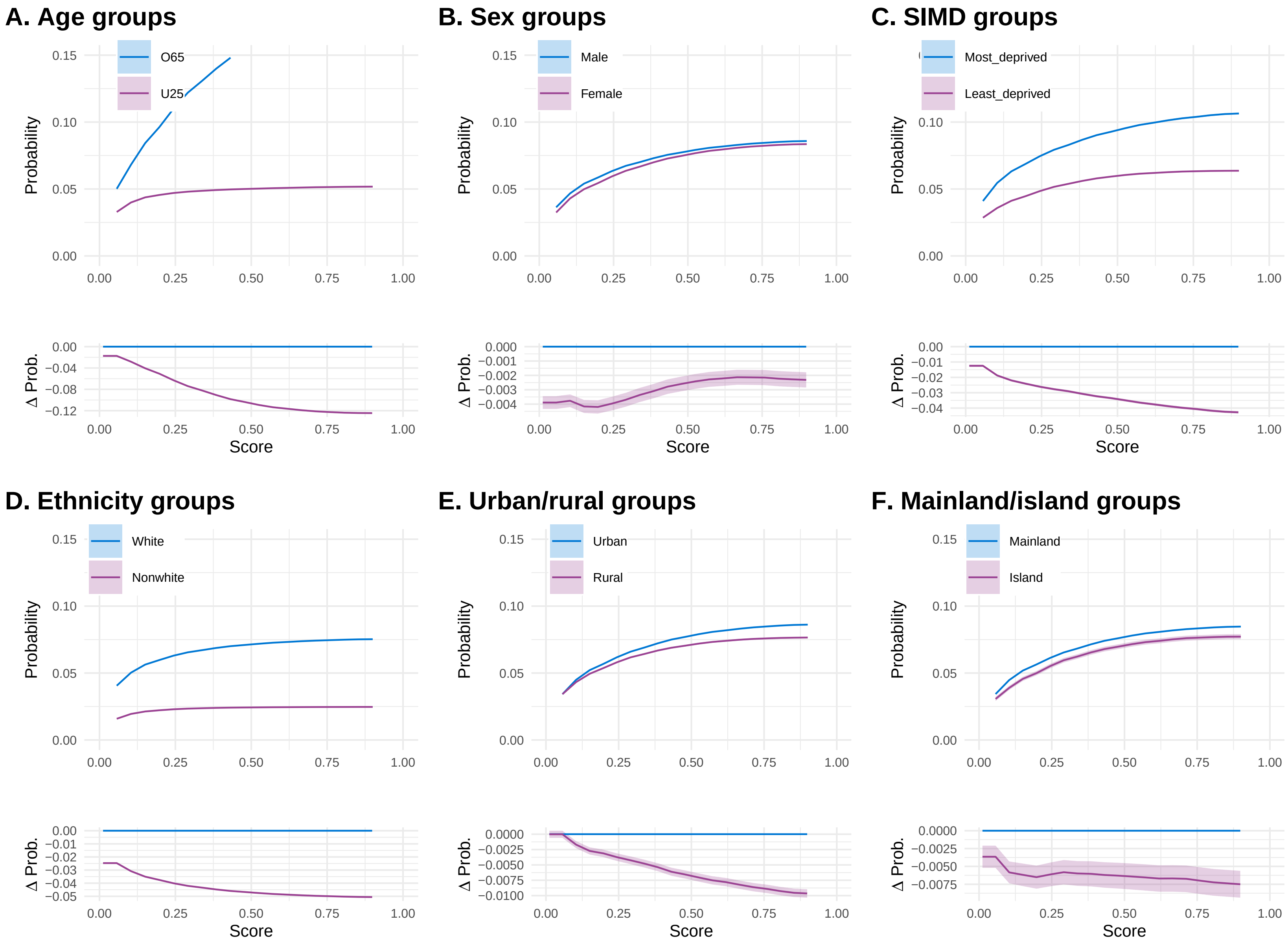
A. Age groups
O65
U25
B. Sex groups
Male
Female
C. SIMD groups
Most_deprived
Least_deprived
D. Ethnicity groups
White
Nonwhite
E. Urban/rural groups
Urban
Rural
F. Mainland/island groups
Mainland
Island
Probability
Δ Prob.
Score
0.00
0.05
0.10
0.15
0.25
0.50
0.75
1.00
−0.04
−0.08
−0.12
0.000
−0.001
−0.002
−0.003
−0.004
−0.01
−0.02
−0.03
−0.05
0.0000
−0.0025
−0.0050
−0.0075
−0.0100
