# Supplementary material for: Differential behaviour of a risk score for emergency hospital admission by demographics in Scotland—A retrospective study
Source: PLOS Digit Health. 2024 Dec 17;3(12):e0000675. doi: 10.1371/journal.pdig.0000675 (PMC11651550; doi:10.1371/journal.pdig.0000675)
Supplement: S7 Fig — Lower sub-panels on each panel show difference between curves. Coloured bands show pointwise 95% confidence intervals. Vertical red dashed lines identify a score of 10%. (PDF) [file pdig.0000675.s009.pdf]

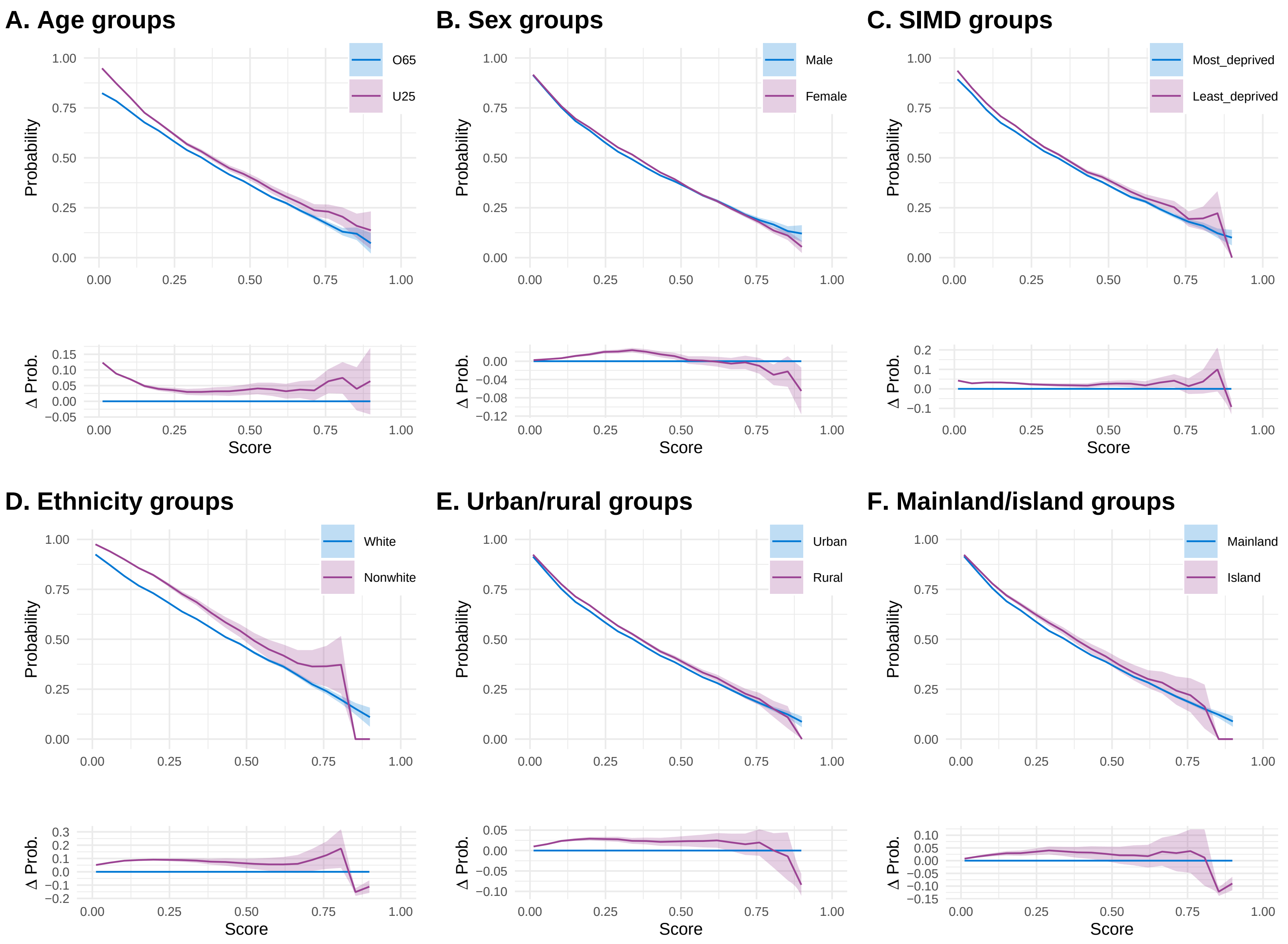
A. Age groups
O65
U25
B. Sex groups
Male
Female
C. SIMD groups
Most_deprived
Least_deprived
D. Ethnicity groups
White
Nonwhite
E. Urban/rural groups
Urban
Rural
F. Mainland/island groups
Mainland
Island
Probability
Δ Prob.
Score
