# Supplementary material for: Differential behaviour of a risk score for emergency hospital admission by demographics in Scotland—A retrospective study
Source: PLOS Digit Health. 2024 Dec 17;3(12):e0000675. doi: 10.1371/journal.pdig.0000675 (PMC11651550; doi:10.1371/journal.pdig.0000675)
Supplement: S8 Fig — Lower sub-panels on each panel show difference between curves. Coloured bands show pointwise 95% confidence intervals. Vertical red dashed lines identify a score of 10%. (PDF) [file pdig.0000675.s010.pdf]

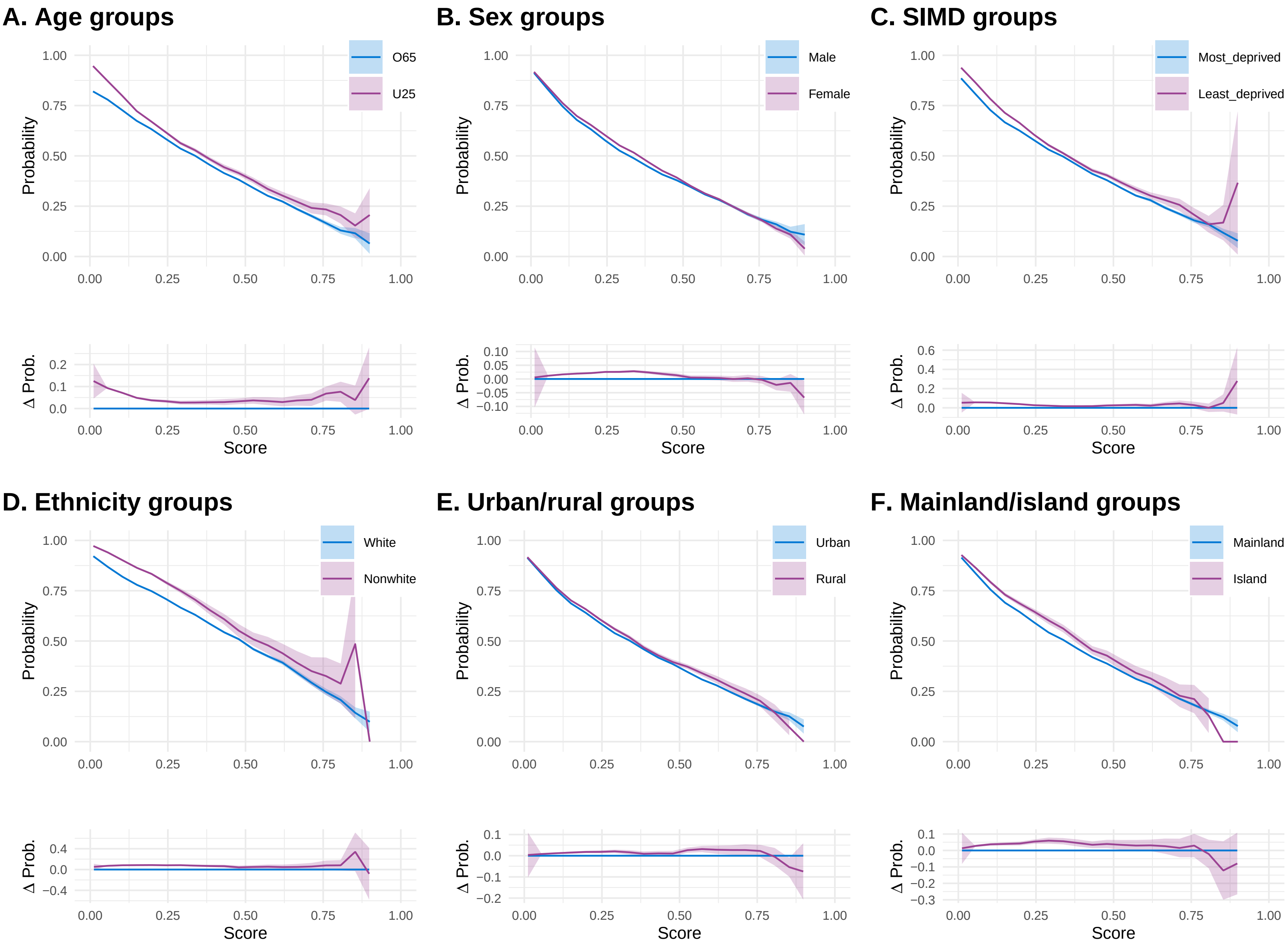
A. Age groups
B. Sex groups
C. SIMD groups
D. Ethnicity groups
E. Urban/rural groups
F. Mainland/island groups
O65
U25
Male
Female
Most_deprived
Least_deprived
White
Nonwhite
Urban
Rural
Mainland
Island
Probability
Δ Prob.
Score
0.00
0.25
0.50
0.75
1.00
0.0
0.1
0.2
0.10
0.05
0.00
−0.05
−0.10
0.2
0.4
0.6
0.4
0.0
−0.4
0.1
0.0
−0.1
−0.2
−0.3
