# Supplementary figures and images for: Differential behaviour of a risk score for emergency hospital admission by demographics in Scotland—A retrospective study
Source: PLOS Digit Health. 2024 Dec 17;3(12):e0000675. doi: 10.1371/journal.pdig.0000675 (PMC11651550; doi:10.1371/journal.pdig.0000675)

Group: All

(Freq. with score < 0.1) - (Freq. in all adm.)

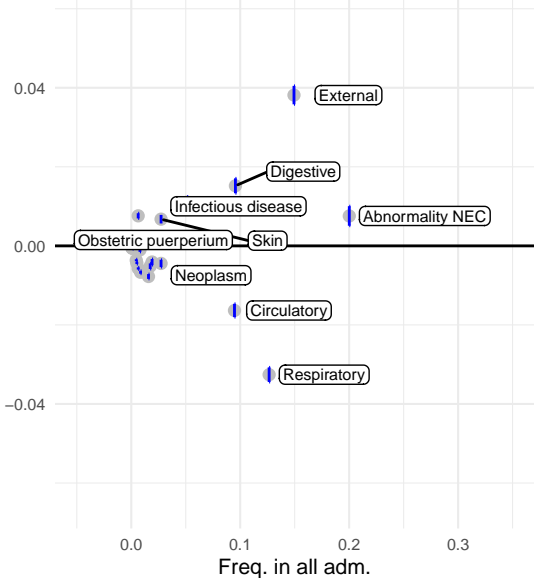

Supplement: S9 Fig — Plots consider the proportion of each admission type amongst all admissions in a group (A) and the proportion of each admission type amongst admissions in the group with SPARRA score < 10% (B) and plots show (A) against (B) − (A). Points above the line y = 0 correspond to admissions which are disproportionately poorly identified by SPARRA score (strictly the criterion Y^<0.1)., Blue vertical lines show pointwise 95% confidence intervals. Upper plots show the proportion of (unpredicted) admissions due to each cause; lower plots show the proportion of deaths due to each cause. Distinctive points are labelled. (PDF) [file pdig.0000675.s011.pdf]

**A. Group: Most\_deprived**

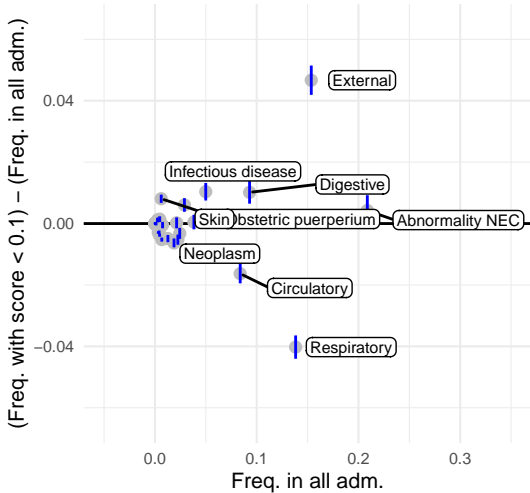

**B. Group: Least\_deprived**

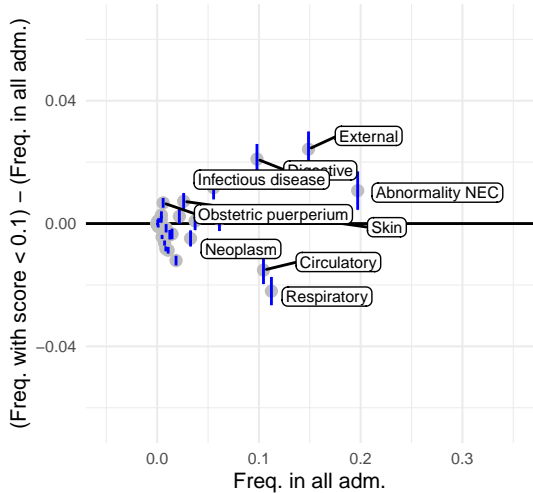

**C. Group: Rural**

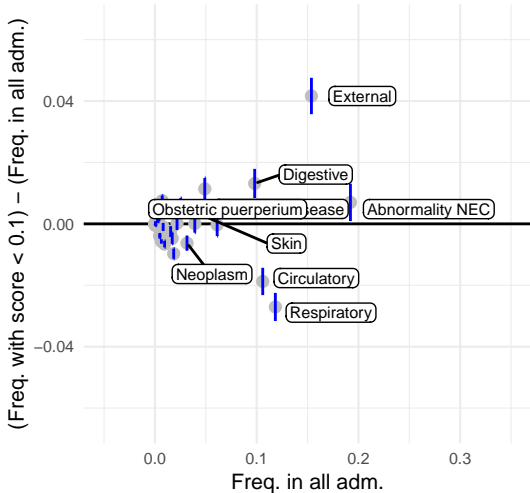

**D. Group: Urban**

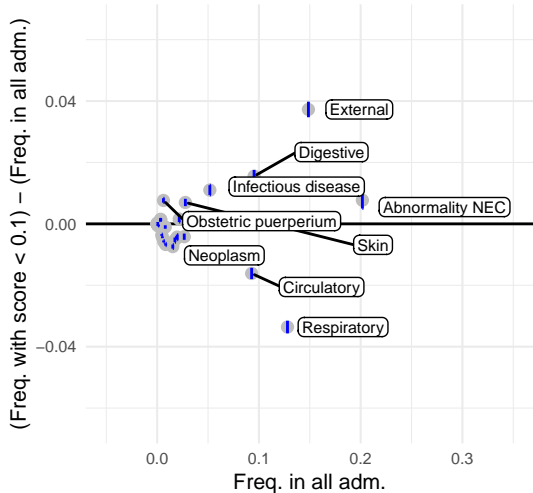

Supplement: S10 Fig — Plots consider the proportion of each admission type amongst all admissions in a group (A) and the proportion of each admission type amongst admissions in the group with SPARRA score < 10% (B) and plots show (A) against (B) − (A). Points above the line y = 0 correspond to admissions which are disproportionately poorly identified by SPARRA score (strictly the criterion Y^<0.1)., Blue vertical lines show pointwise 95% confidence intervals. Upper plots show the proportion of (unpredicted) admissions due to each cause; lower plots show the proportion of deaths due to each cause. Distinctive points are labelled. (PDF) [file pdig.0000675.s012.pdf]

**A. Group: White**

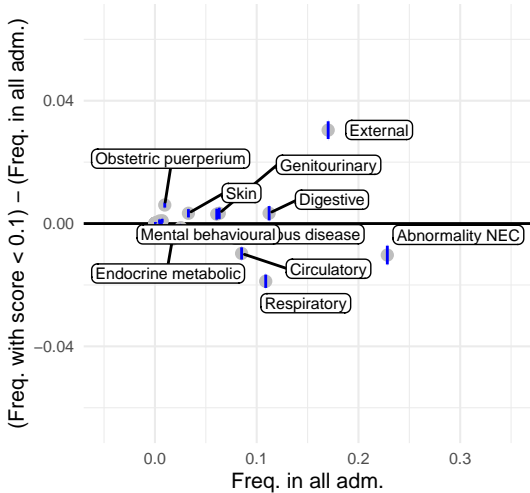

**B. Group: Nonwhite**

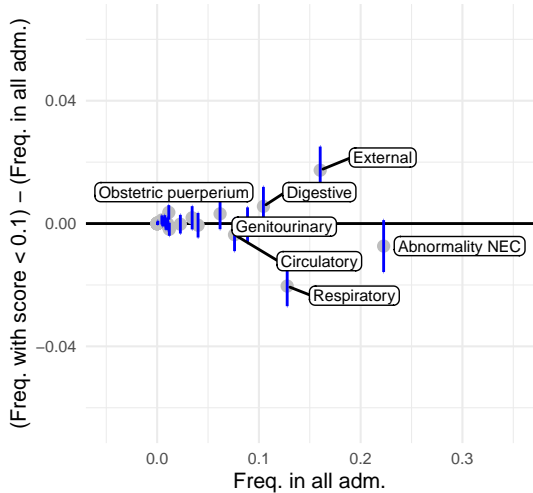

**C. Group: U25**

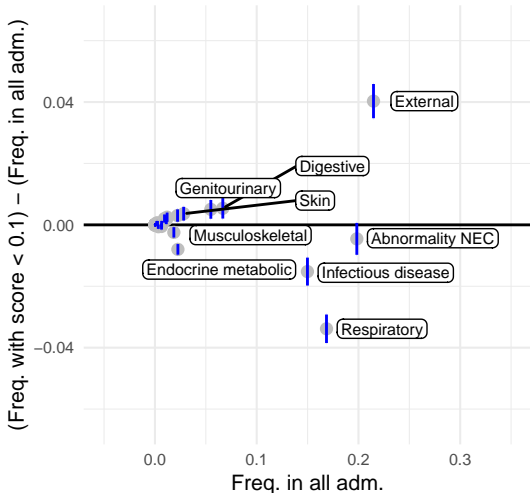

**D. Group: O65**

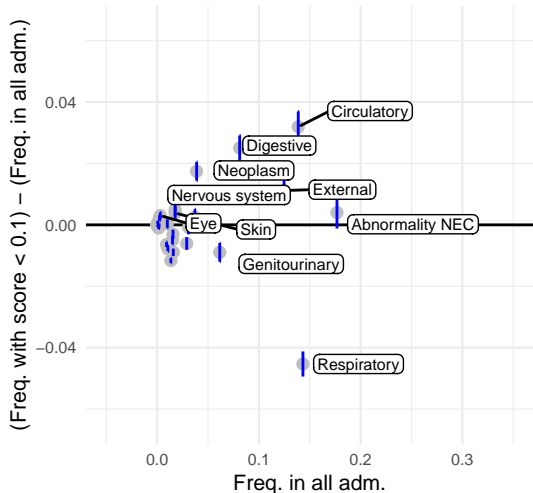

Supplement: S11 Fig — Plots consider the proportion of each admission type amongst all admissions in a group (A) and the proportion of each admission type amongst admissions in the group with SPARRA score < 10% (B) and plots show (A) against (B) − (A). Points above the line y = 0 correspond to admissions which are disproportionately poorly identified by SPARRA score (strictly the criterion Y^<0.1)., Blue vertical lines show pointwise 95% confidence intervals. Upper plots show the proportion of (unpredicted) admissions due to each cause; lower plots show the proportion of deaths due to each cause. Distinctive points are labelled. (PDF) [file pdig.0000675.s013.pdf]

## A. Group: Mainland

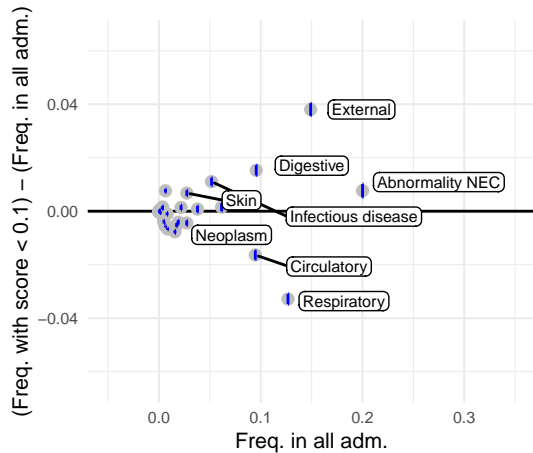

## B. Group: Island

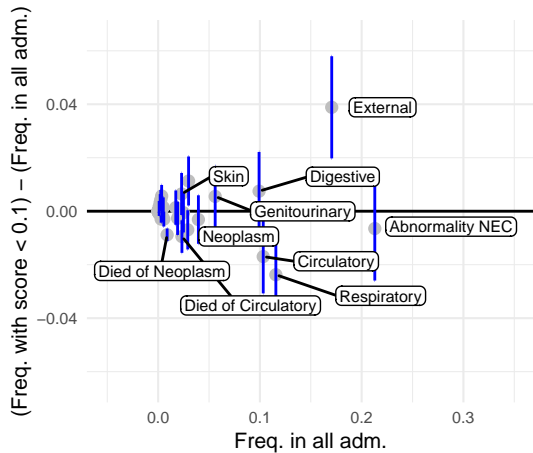

Supplement: S12 Fig — Plots consider the proportion of each admission type amongst all admissions in a group (A) and the proportion of each admission type amongst admissions in the group with SPARRA score < 10% (B) and plots show (A) against (B) − (A). Points above the line y = 0 correspond to admissions which are disproportionately poorly identified by SPARRA score (strictly the criterion Y^<0.1)., Blue vertical lines show pointwise 95% confidence intervals. Upper plots show the proportion of (unpredicted) admissions due to each cause; lower plots show the proportion of deaths due to each cause. Distinctive points are labelled. (PDF) [file pdig.0000675.s014.pdf]
