# Supplementary material for: Differential behaviour of a risk score for emergency hospital admission by demographics in Scotland—A retrospective study
Source: PLOS Digit Health. 2024 Dec 17;3(12):e0000675. doi: 10.1371/journal.pdig.0000675 (PMC11651550; doi:10.1371/journal.pdig.0000675)
Supplement: Attachment — Submitted filename: rebuttal_letter.pdf [file pdig.0000675.s015.pdf]

Response to reviewer comments on ‘Differential behaviour of a risk score for emergency hospital admission by demographics in Scotland — a retrospective study’, by Thoma, Rogers, Ireland, et al.

General comments to editor and to both reviewers

We thank both reviewers and the editor for their careful reading and insightful comments. We have responded specifically to each reviewer below. We have coloured changed text blue in the main manuscript.

Comments from reviewers remain in their original order. We have added an enumeration scheme: A1, A2, . . . for reviewer 1; B1, B2, . . . for reviewer 2.

Our own comments

We have made occasional corrections to typographic errors when we have seen them. We have also corrected an error in SR’s affiliation.

Reviewer 1

> General comments: Excellent paper, well-written and with interesting results. Only two minor comments as shown below:

We thank the reviewer for these comments, and are pleased that they consider our paper well-written and the results interesting.

> A1: Clarify how does the SPARRA scoring system works.

We apologise for our lack of clarity. We have made the following changes to our manuscript which we hope will make this clearer.

1. We have added a sentence to the second paragraph in the ‘Introduction’ indicating that SPARRA scores range from 1 to 99, and that higher scores indicate a higher estimated probability of admission. Our added sentence reads ‘SPARRA scores range from 1 to 99, with higher scores indicating a higher estimated probability of admission.’

2. Also in the second paragraph of the 'Introduction', we have added a citation to a website *SPARRA made easy*, intended as a user guide for non-experts. The reference also provides a link to the various SPARRA case studies.

3. We have added a note to the second paragraph of the 'Introduction' section indicating that general practitioners have previously been advised to review patients with SPARRA scores in the range 40-60, with the intention of identifying patients not already known to be at high risk of emergency admission. Our new note reads:

    > For example, GPs have previously been incentivised to review patients with SPARRA scores in the range 40-60, with the intention of identifying patients not already known to be at high risk of emergency admission.

> A2: Add the strengths and limitations at the end of the discussion section

We thank the reviewer for this suggestion. We have added a paragraph to the 'Discussion' section (paragraph 2) where we have said the following:

> One of the key advantages of this study is that it uses national-level data that reflect a real-world situation and the implications of a model that has been deployed in healthcare settings for many years. Furthermore, we used multiple fairness metrics to attain comprehensive insight into the differences in scores and error rates between population subgroups. Our online dashboard enables researchers, practitioners and members of the public to explore these results interactively, providing detailed information about the performance of SPARRA.

We have also added a sentence at the beginning of paragraph 5 in the 'Discussion' section, which indicates that we consider the content of this paragraph to concern the limitations of our study. We have also highlighted an additional potential limitation to this paragraph, reading as follows:

> One limitation of this study that should be considered when interpreting the results is that this study looked exclusively at routinely collected historical data, which may be subject to a variety of observational biases. [...] Furthermore, our work did not consider how SPARRA is integrated into the healthcare system and did not involve the analysis of healthcare decisions (e.g. primary care interventions informed by SPARRA). As such, we are not able to identify disadvantaged groups or inequity in healthcare provision, nor recommend any changes to the distribution of healthcare resources in Scotland or to the way the SPARRA score is used. However, our findings may facilitate ongoing analyses in these areas.

Please also see our response to comment B4, in which we identify that the ratio of white- to non-white individuals in our dataset does not match that of the general population.

Reviewer 2

> General comments: Very good study with important findings. Some clarifications are needed as below:

We thank the reviewer for their comments and are pleased with their evaluation of our study as containing important findings.

B1: In the abstract, state briefly what are the causes that led to false positive and false negative predictions.

We have modified the abstract to contain the following sentence:

> We show that the score is well-calibrated across subgroups, but that rates of false positives and negatives vary widely, mediated by various causes including variability in demographic characteristics, admission reasons, and potentially differential data availability.

B2: The importance of equitable predictions and the need for them should be highlighted further in the introduction section.

We thank the reviewer for this suggestion. We do consider that, in light of the many ways to consider differential behaviour of the SPARRA score in our paper, there are similarly many potential ways to define equitability. However, we agree with the general idea of the reviewer that it is vitally important that the SPARRA score contribute to equitable healthcare.

In the third paragraph of the 'Introduction' section, we have added the following sentence:

> A major motivation for our work was to ensure that the SPARRA score did not worsen equity in healthcare provision. We aimed to partly assess this by analysing several conceptions by which the score could in itself be considered to be making equitable predictions.

B3: In the methods, justify the inclusion from 1981 onwards. Why were ICD-10 codes used and not ICD-11?

To clarify the inclusion of data from 1981 onwards, we have added the following sentences to the second paragraph of Data in Materials and Methods:

> [...] and long-term condition (LTC) records dating back to January 1981 (when records began) to capture any long-term conditions an individual has acquired throughout their life.

Furthermore, the following sentence regarding the use ICD-10 codes has been added to Metrics in Material and Methods:

> Our study spans the years 2013-2018, prior to the publication of ICD-11

B4: The ratio of white to non-white is higher. Can the authors comment on this in more details in the discussion.

We recognise that there is non-random missingness in our ethnicity data, and that the overall frequency of non-missing ethnicity values in our sample is not typical of the general Scottish population.

The causes for this are likely to be complex and we do not feel we can reasonably comment on them based on the data we have available. However, we do agree with the reviewer that this observation is important, and we specifically note in the discussion section that our findings related to ethnicity must be interpreted in light of the presence of non-random missingness. Specifically, we say:

> In particular, the interpretation of ethnicity data is complex due to non-random missingness (see Supplementary Section *Ethnicity determination*): in particular, our study cohort has a higher ratio of people identifying as white to people identifying as non-white than does the population of Scotland as a whole. Our findings related to ethnicity must be interpreted in light of this missingness.

Please also see our response to comment A2.
